# Circulating mature dendritic cells homing to the thymus promote thymic epithelial cells involution via the Jagged1/Notch3 axis

**DOI:** 10.1038/s41420-021-00619-5

**Published:** 2021-08-30

**Authors:** Haojie Wu, Xiaohan Li, Chen Zhou, Qihong Yu, Shiyao Ge, Zihui Pan, Yangjing Zhao, Sheng Xia, Xiaoming Zhou, Xia Liu, Hui Wang, Qixiang Shao

**Affiliations:** 1 Reproductive Sciences Institute, Jiangsu Key Laboratory of Medical Science and Laboratory Medicine, Department of Immunology, School of Medicine, Jiangsu University, Zhenjiang, 212013 Jiangsu P. R. China; 2 Jiangsu Key Laboratory of Medical Science and Laboratory Medicine, Department of Pathology, School of Medicine, Jiangsu University, Zhenjiang, 212013 Jiangsu P. R. China; 3 Institute of Medical Genetics and Reproductive Immunity, School of Medical Science and Laboratory Medicine, Jiangsu College of Nursing, Huai’an, 223002 Jiangsu P. R. China

**Keywords:** Cell death and immune response, Immune cell death, Signal transduction

## Abstract

Multiple proinflammatory conditions, including chemotherapy, radiotherapy, transplant rejection, and microbial infections, have been identified to induce involution of the thymus. However, the underlying cellular and molecular mechanisms of these inflammatory conditions inducing apoptosis of thymic epithelial cells (TECs), the main components of the thymus, remain largely unknown. In the circulation, mature dendritic cells (mDCs), the predominant initiator of innate and adaptive immune response, can migrate into the thymus. Herein, we demonstrated that mDCs were able to directly inhibit TECs proliferation and induce their apoptosis by activating the Jagged1/Notch3 signaling pathway. Intrathymic injection of either mDCs or recombinant mouse Jagged1-human Fc fusion protein (rmJagged1-hFc) into mice resulted in acute atrophy of the thymus. Furthermore, DAPT, a γ-secretase inhibitor, reversed the effects induced by mDC or rmJagged1-hFc. These findings suggest that acute or aging-related thymus degeneration can be induced either by mass migration of circulating mDCs in a short period of time or by a few but constantly homing mDCs.

## Introduction

The thymus, as a central immune organ, is mainly responsible for T lymphocytes production. Unlike other organs that begin to develop after birth, the thymus begins to degenerate from the first year of life in humans [1]. Thymic atrophy is caused by physiological aging, and diseases (pathological conditions) such as severe acute respiratory syndrome coronavirus 2 (SARS-CoV-2) [2], severe HIV infection [3], transplant rejection, radiotherapy, and chemotherapy [4]. The thymic microenvironment is composed of thymic stromal cells, extracellular matrix, cytokines, peptides, and hormones [5, 6]. As a predominant component of thymic stromal cells, thymic epithelial cells (TECs) govern the positive and negative selection of T cells, and promote the maturation of T cells. The degeneration of TECs causes alterations in the thymic microenvironment that result in the dysfunction of T-cell production, thus reducing their irreversible output, TCR repertoire, and increasing susceptibility to severe infections such as COVID-19 [7]. Furthermore, TECs degradation can disrupt central T-cell immune tolerance, cause hyperreactivity to self-antigens, and be easy to suffer from autoimmune diseases [8]. Thus, elucidating the mechanisms underlying thymic involution is essential for reversing thymic atrophy, restoring thymic function, and promoting T-cell development.

Both the increase in sex steroids and the decrease of growth hormones induce thymic involution with aging [9]. Several signaling pathways, including Notch signaling [10], Wnt/β-catenin [11], and bone morphogenetic protein (BMP) signaling [12], are also involved in thymic development. Notch signaling plays critical roles in cell development, homeostasis, and disease processes, such as stem cell renewal, embryonic and organic development, cardiomyopathy, oncogenesis, etc [13]. In mammals, the Notch signaling pathway is composed of receptors (Notch1-4), ligands (DLL1/3/4 and Jagged-1/2), and CSL (CBF1/suppressor of hairless/lag-1, also known as immunoglobulin kappa J region recombination signal binding protein 1 (RBP-1/RBP-Jκ)) [14, 15]. Notch signaling occurs through cell–cell interaction where transmembrane ligands present on one cell activate transmembrane receptors present on another cell. Ligand-mediated Notch signaling activation induces the proteolytic cleavage of receptors, resulting in the release of the Notch intracellular domain (NICD). Subsequently, the NICD translocates into the nucleus, forms a complex with the DNA-binding protein CSL and the coactivator mastermind, and then initiates the transcription of target genes [13, 14, 16].

Previous studies showed that the Notch signaling is essential for maintaining thymic epithelial progenitor cells and medullary epithelial cells in fetal thymic development [10]. Mice carrying mutations in Jagged2, one of the ligands of Notch receptors, exhibited impaired thymic development and morphology [17]. In contrast, Beverly et al. reported that the overexpression of Jagged1 induced the apoptosis of TECs in T-lymphocyte Jagged1-transgenic mice [18]. Histone deacetylase 3-mediated repression of Notch signaling is critical for medullary thymic epithelial cells (mTECs) development [19]. TEC-specific overexpression of Notch1 reduced the populations of total TECs and mTECs, suggesting that Notch signaling promotes the degeneration of the mTEC lineage [19]. Taken together, these findings indicate that Notch signaling plays complex roles in thymic development and degeneration. However, its underlying mechanisms remain unclear.

Dendritic cells (DCs) are critical antigen-presenting cells. Exposed to dangerous signals, the tissue-resident DCs upregulate the expression of costimulatory molecules, migrate into local lymph nodes, and induce the proliferation and differentiation of naïve T cells into effector T cells [20–22]. In addition, by increasing the expression of costimulatory molecules, DCs induce regulatory T cells (Tregs) expansion and suppress Th2 response through Jagged1 expression [23, 24]. Furthermore, natural killer (NK) cells are activated by DCs through the Jagged2-Notch interaction [25]. These results suggest that the Notch signaling is critical in mediating the interactions between DCs and other immune cells. Additionally, peripheral DCs can migrate into the thymus and deplete thymocytes [26]. Hence, in this study, we examined whether circulating DCs migrated into the thymus and induced the degeneration of TECs via Notch signaling.

## Results

### Mature DCs migrate into the thymus

**Fig. 1:**
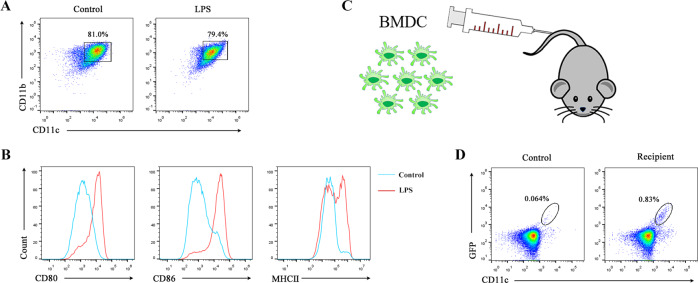
Mature DCs could return to the thymus. **A** Bone marrow cells were isolated and cultured with GM-CSF (10 ng/ml) plus IL-4 (1 ng/ml) for 7 days. On day 7, imBMDCs were harvested and incubated with LPS (1 μg/ml). Subsequently, the cells were collected and stained with anti-CD11c and anti-CD11b antibodies. The expressions of CD11c and CD11b in BMDCs were assessed by FCM. **B** BMDCs were stained with anti-CD11c, anti-CD11b, anti-CD80, anti-CD86, and anti-MHC II antibodies. The expressions of CD80, CD86, and MHC II in CD11c^+^ CD11b^+^ cells were verified by FCM. **C** The carton showed that GFP^+^ mBMDCs (1.0 × 10^7^) were injected into wild-type mice via the caudal vein. **D** CD11c^+^ GFP^+^ cells from the thymus of recipients were analyzed by FCM. All data are from three independent experiments. Representative figures of these three experiments are shown.

Upon interactions with pathogens or their products (e.g., LPS), circulating and tissue-resident DCs were activated and underwent maturation processes [22]. To examine whether mature DCs (mDCs) were able to return to the thymus, we isolated bone marrow-derived mononuclear cells from GFP-transgenic (EGFP^Tg/+^) mice to generate bone marrow-derived DCs (BMDCs) in the presence of granulocyte-macrophage colony-stimulating factor (GM-CSF) plus interleukin (IL)-4 in vitro (Fig. 1A). The results revealed that the expression levels of costimulatory molecules, including CD80, CD86, and MHC II, evaluated by flow cytometry (FCM), were upregulated on BMDCs after LPS treatment (Fig. 1B). Subsequently, the mDCs were harvested and injected into wild-type mice via the lateral tail vein (Fig. 1C). The thymuses of the recipient mice were isolated 3 days later and GFP ^+^ cells were detected by FCM. Almost all GFP^+^ cells were CD11c positive (Fig. 1D). These results indicated that circulating mDCs were able to migrate into the thymus.

### Mature DCs promoted cell apoptosis and inhibited TECs proliferation through cell–cell contact

**Fig. 2:**
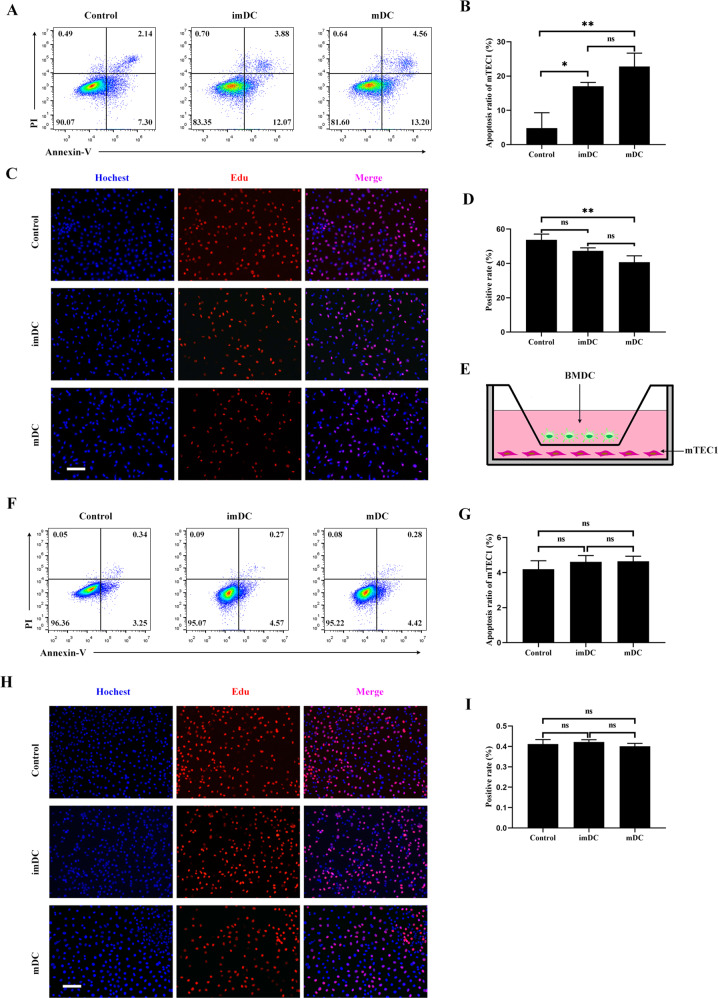
DCs induced the apoptosis and inhibited the proliferation of mTEC1 cells through direct cell–cell contact. **A** mTEC1 cells were cocultured with imBMDCs or mBMDCs at a 1:3 ratio for 48 h, and cell apoptosis was examined through Annexin V/PI staining with FCM. mTEC1 cells alone were set as the control group. **B** The percentages of apoptotic cells in different groups were determined according to panel **A**. **C** mTEC1 cells were cocultured with imBMDCs or mBMDCs at a 1:3 ratio for 48 h, and cell proliferation was examined using an EdU incorporation assay. Red fluorescent dots indicate proliferating cells. Scale bars represent 100 µm. **D** The percentages of red fluorescent dots in all cells were calculated according to panel **C**. **E** Sketch map of the transwell system. mBMDCs were seeded in the upper well, and mTEC1 cells were seeded at the bottom chamber under the membrane. **F** mTEC1 cells were cocultured with imBMDCs or mBMDCs in the transwell system for 48 h. Cell apoptosis was examined through Annexin V/PI staining by FCM assay. **G** The percentages of apoptotic cells in different groups were examined according to panel **F**. **H** mTEC1 cells and imBMDCs or mBMDCs were cocultured in the transwell system for 48 h. Cell proliferation was examined by using an EdU incorporation assay. Red fluorescent dots represent proliferating cells. Scale bars represent 200 µm. **I** The percentages of red fluorescent dots in all cells were computed according to panel **H**. All data are from three independent experiments. Representative figures of these three experiments are shown. Data are represented as the mean ± SD. *P < 0.05, **P < 0.01, compared with the control group.

To examine the effects of homing DCs on TECs, we cocultured the thymic epithelial cell line mTEC1 with immature BMDCs (imBMDCs) or mature BMDCs (mBMDCs). Compared to the control group, both imBMDCs and mBMDCs induced the apoptosis of mTEC1 cells, whereas mBMDCs showed a stronger effect than imBMDCs (Fig. 2A, B). The EdU incorporation assay demonstrated that both imBMDCs and mBMDCs were able to inhibit the proliferation of mTEC1 cells, while the inhibiting ability of mBMDCs was more effective than that of imBMDCs (Fig. 2C, D). Furthermore, we performed a transwell coculture assay to investigate how BMDCs affect mTEC1 cells (Fig. 2E). The ratio of apoptosis and proliferation of mTEC1 cells had no significant changes in the transwell system (Fig. 2F–I). These results imply that mDCs/mTEC1 physical cell–cell contact is essential for DC-mediated involution of mTEC1 cells.

### Jagged1 was highly expressed in mDCs, while Notch3 was predominantly present in mTECs

**Fig. 3:**
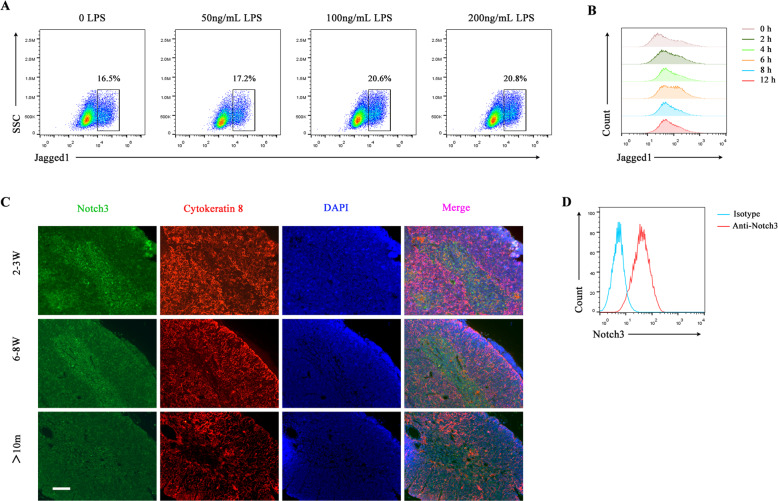
Mature BMDCs expressed a high level of Jagged1, whereas mTECs expressed Notch3. **A** BMDCs were stained with the anti-Jagged1 antibody. The expression level of Jagged1 in BMDCs was examined using FCM after stimulation with different concentrations of LPS for 6 h. **B** The expression level of Jagged1 in BMDCs after stimulation with LPS (100 ng/ml) for different durations was detected using FCM. **C** Representative images of the expression of Notch3 (green fluorescence) and cytokeratin 8 (K8, a cTEC marker, red fluorescence) in the thymus from different aged mice (2–3 weeks old, 6–8 weeks old, and 10 months old, respectively) were captured by immunofluorescence microcopy. Paraffin sections of the thymus obtained from different aged mice were stained with primary rabbit anti-Notch3 and rat anti-cytokeratin 8 antibodies, followed by secondary Alexa Fluor 488-labeled goat anti-rabbit and Alexa Fluor 555-labeled goat anti-rat antibodies, respectively. Finally, the sections were stained with DAPI. Scale bars represent 200 µm. **D** mTEC1 cells were stained with PE-conjugated anti-Notch3 antibody. The expression of Notch3 in mTEC1 cells was detected by FCM. All data are from three independent experiments. Representative figures of these three experiments are displayed.

Previous studies have shown that overexpression of Jagged1 on thymocytes can induce apoptosis of thymic stromal epithelial cells in T-lymphocyte Jagged1-transgenic mice, leading to thymic atrophy [18]. Thus, we would like to know whether the mBMDCs expressed a higher level of Jagged1 compared with imBMDCs. We treated imBMDCs with different concentrations and durations of LPS and found that the expression of Jagged1 present on the surface of BMDCs was upregulated after LPS stimulation in a dose- and time-dependent manner. The highest level of Jagged1 expression was observed after stimulation with 100 ng/ml LPS for 6 h (Fig. 3A, B). In addition, we also demonstrated that Notch3 was mainly expressed in the medullary region of the thymus and its level decreased during the aging process (Fig. 3C). Moreover, Notch3 expression was noted in mTEC1 cells (Fig. 3D). Thus, mDCs expressed Jagged1, the Notch ligand, and mTECs expressed Notch3, the Notch receptor. These results suggest that mDCs might promote thymus degeneration through the Notch signaling.

### MDCs induced apoptosis and inhibited proliferation of mTEC1 cells by activating Notch signaling

**Fig. 4:**
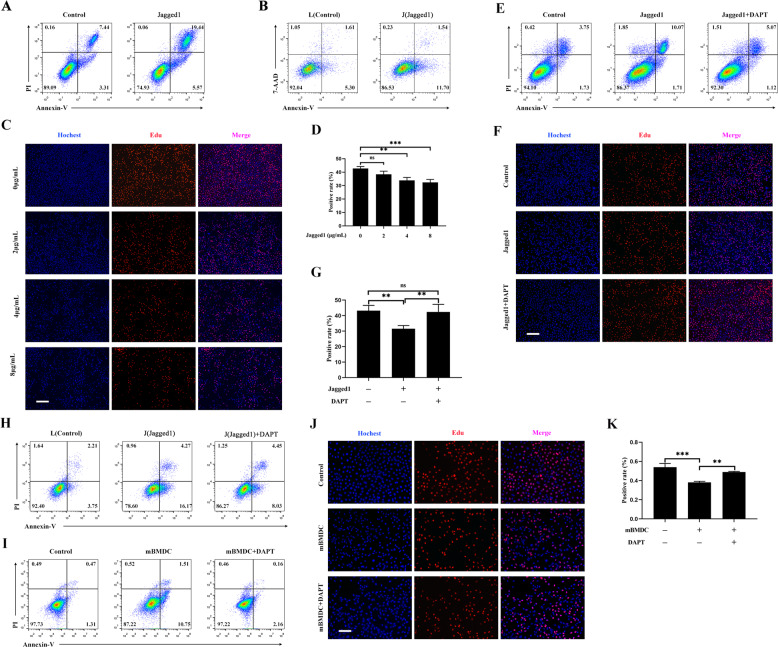
Mature DCs promoted the apoptosis and inhibited the proliferation of mTEC1 cells by activating Notch signaling. **A** rmJagged1-hFc was precoated on a 24-well plate at 4 °C for 12 h. BSA precoated at the same concentration was used as the control. mTEC1 cells were seeded at a density of 6 × 10^4^ cells per well. Cell apoptosis was examined through staining with Annexin V/PI. **B** L and J cells were pre-stained with CFSE. mTEC1 cells were then cocultured with L or J cells at a 1:3 ratio for 48 h. The apoptosis of mTEC1 cells was examined through staining with Annexin V-APC/7-AAD. **C** The different concentrations of rmJagged1-hFc were precoated on 96-well plates at 4 °C for 12 h. The same concentration of BSA was set as the control group. mTEC1 cells were seeded at a density of 5 × 10^3^ cells per well. Cell proliferation was examined using an EdU incorporation assay after 48 h. The red fluorescent dots indicate proliferating cells. Scale bars represent 200 µm. **D** The percentages of red fluorescent dots in all cells were calculated according to panel **C**. **E** Experiments were performed as described in **A**. mTEC1 cells were treated with or without DAPT (10 μM). Cell apoptosis was analyzed through Annexin V/PI staining. **F** Experiments were performed as described in **C**. Proliferation was detected by performing an EdU incorporation assay. mTEC1 cells were incubated with or without DAPT (10 μM). Red fluorescent dots indicate proliferating cells. Scale bars represent 200 µm. **G** The percentages of red fluorescent dots in all cells were calculated according to panel **F**. **H** Experiments were performed as described in **B**. mTEC1 cells were treated with or without DAPT (10 μM). **I** Experiments were performed as shown in Fig. 2A. mTEC1 cells were cultured with or without DAPT (10 μM). **J** Experiments were performed as shown in Fig. 2C. mTEC1 cells were treated with or without DAPT (10 μM). Scale bars represent 200 µm. **K** The percentages of red fluorescent dots in all cells were calculated according to panel **J**. All data are from three independent experiments. Representative figures of these three experiments are shown. Data are expressed as the mean ± SD. **P < 0.01, ***P < 0.001 compared with the control group.

To determine whether Notch signaling played a critical role in thymic involution, mTEC1 cells were boosted with coating rmJagged1-hFc to activate Notch signaling. The results declared that Jagged1 protein accelerated the apoptosis of mTEC1 cells (Fig. 4A). Next, we cocultured a Jagged1 stably transfected fibroblast cell line (J cells) with mTEC1 cells and used a fibroblast cell line without Jagged1 expression (L cells) as a control. We found that the percentage of apoptotic mTEC1 cells increased after cocultured with J cells (Fig. 4B). Meanwhile, Jagged1 protein suppressed the growth of mTEC1 cells (Fig. 4C, D). Next, we used DAPT, an inhibitor of γ-secretase, to block the signal transduction of the Notch signaling. As we expected, DAPT reversed the cell apoptosis and the inhibition of cell proliferation induced by rmJagged1-hFc (Fig. 4E–G). All these findings suggested that activated Notch signaling resulted in the apoptosis and growth inhibition of mTEC1 cells. To further validate if mDCs affect mTEC1 cells through Notch signaling, we added DAPT to the mTEC1/J cell coculture system. Our results showed that DAPT reversed the apoptosis of mTEC1 cells induced by J cells (Fig. 4H). In addition, DAPT also attenuated the apoptosis and proliferative inhibition of mTEC1 cells induced by BMDCs (Fig. 4I–K). Hence, these results illustrated that mDCs induced apoptosis and inhibited proliferation of mTEC1 cells via activating the Notch signaling.

### Intrathymic injection of mature DCs led to acute thymic atrophy

**Fig. 5:**
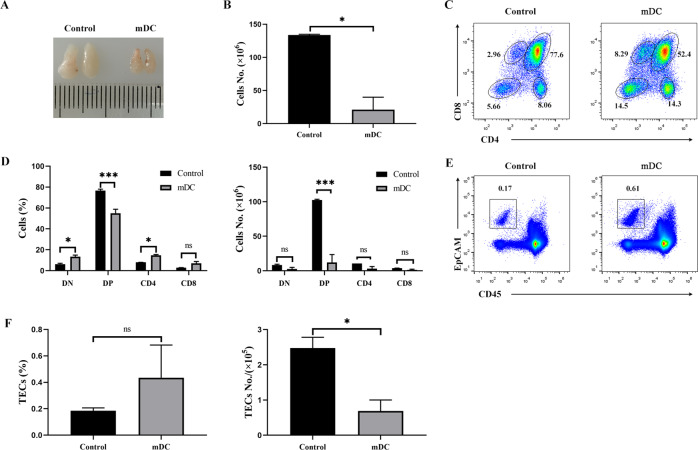
Intrathymic injection of mature DCs led to acute thymic atrophy. For **A** to **F**: Thymic atrophy following the intrathymic injection of mDCs (*n* = 3). The same volume of PBS was used as a control (*n* = 3). **A** Thymus morphology and size. **B** Total numbers of thymocytes were calculated. **C** Thymocytes were stained with PE-conjugated anti-CD4 and APC-labeled anti-CD8 antibodies. The thymocyte subpopulations were examined by FCM. **D** The percentages and absolute numbers of indicated thymocyte subpopulations were calculated. **E** The cells isolated from the thymus of recipient mice were stained with Alexa Fluor 488-conjugated anti-CD45 and APC-labeled anti-EpCAM antibodies, respectively. CD45^-^EpCAM^+^ TECs were detected by FCM. **F** The percentages and absolute numbers of CD45^-^EpCAM^+^ TECs were calculated. All data are from three independent experiments. Representative figures of these three experiments are shown. Data are expressed as the mean ± SD. *P < 0.05, ***P < 0.001 compared with the control group.

As shown above, the circulating mDCs were able to migrate into the thymus. However, the number of homing DCs was considerably a few. Moreover, it was difficult to evaluate or observe the effect of homing mDCs on the thymus in a short period time. Thus, to investigate the effect of mDCs on TECs, the mDCs were intrathymically injected into 6–8-week-aged mice. After 3 days, we observed that the thymic size of the recipient mice was smaller than that of control mice injected with PBS (Fig. 5A). Additionally, we noted that the number of thymocytes decreased significantly in the process (Fig. 5B). The percentage of CD4 ^+^ CD8 ^+^ DP thymocytes was decreased. However, those of CD4^−^ CD8^−^ DN, CD4^+^ SP, and CD8^+^ SP thymocytes were increased (Fig. 5C, D). In contrast, we found a substantial decrease in the absolute numbers of all thymocyte subpopulations (Fig. 5D). Similar to thymocytes, the percentage of CD45^−^ EpCAM^+^ TECs was increased (Fig. 5E, F). Whereas, the absolute numbers of TECs were decreased in mDC-treated mice (Fig. 5F). Taken together, these data imply that it is a constant but slowly cumulative process for mDC-induced atrophy of the thymus.

### Intrathymic injection of rmJagged1-hFc led to acute atrophy of the thymus

**Fig. 6:**
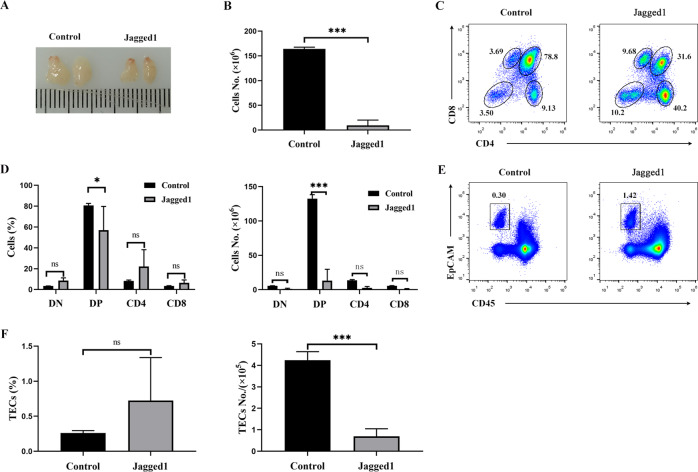
Intrathymic injection of Jagged1 led to thymic atrophy. For **A** to **F**: Thymic atrophy after intrathymic injection of rmJagged1-hFc (*n* = 3). The same volume of PBS was used as a control (*n* = 3). **A** Thymus morphology and size. **B** Total numbers of thymocytes were calculated. **C** Thymocytes were stained with PE-conjugated anti-CD4 and APC-labeled anti-CD8 antibodies. The thymocyte subpopulations were analyzed by FCM. **D** The percentages and absolute numbers of the indicated thymocyte populations were calculated. **E** The cells isolated from the thymus of recipient mice were stained with Alexa Fluor 488-conjugated anti-CD45 and APC-labeled anti-EpCAM antibodies, respectively. CD45^-^EpCAM^+^ TECs were evaluated by FCM. **F** The percentages and absolute numbers of CD45^-^EpCAM^+^ cells were calculated. All data are from three independent experiments. Representative figures of these three experiments are shown. Data are represented as the mean ± SD. *P < 0.05, ***P < 0.001 compared with the control group.

To demonstrate if activated Notch signaling contributed to thymic atrophy, we intrathymically injected the rmJagged1-hFc protein into 6–8-week-aged mice. The thymus was considerably smaller in the treatment group than in the control group after injection (Fig. 6A). We also noted a substantial decrease in total thymocytes numbers (Fig. 6B). The percentages of CD4^−^ CD8^−^ DN, CD4^+^ SP, and CD8^+^ SP thymocytes were increased, while that of CD4^+^ CD8^+^ DP thymocytes was decreased (Fig. 6C, D). The absolute numbers of DP thymocytes were largely decreased. There were no significant differences in the percentage and absolute numbers of DN and SP thymocytes in the rmJagged1-hFc treatment group compared with the control groups. However, the trend of absolute numbers of DN and SP thymocytes was decreased (Fig. 6D). Except for the decrease in thymocytes, the percentage of CD45^−^ EpCAM^+^TECs was increased (Fig. 6E, F), whereas the absolute numbers of TECs were decreased in the rmJagged1-hFc-treated mice (Fig. 6F). Thus, these findings indicate that Jagged1 can lead to the degeneration of the thymus.

## Discussion

The thymus is the central immune organ of the body and critical for T-cell differentiation and development. Many different cell types including thymocytes and thymic stromal cells such as TECs, resident macrophages, and DCs are present in the thymus. As the most crucial stromal cells in the thymus, TECs consist of the cortex and medulla TECs and control the positive and negative selection of T cells [27]. In 1985, Steinmann reported that the volumes of the thymic epithelium (cortex and medulla) show a continuous involution from the first year to the end of life [1]. During thymus degeneration, TECs are replaced by fibrocytes and adipocytes. Decreased thymopoiesis leads to a decreased output of naïve T cells with reduced TCR repertoire and diversity. In addition, the number of naïve T cells in peripheral blood decreases gradually. This process might weaken the protective immune response against pathogens, thus increasing susceptibility to microorganisms such as SARS-CoV-2 [7], and leading to a high incidence of autoimmune disorders in older individuals due to the impairment of negative selection [8].

The increasing evidence demonstrated that peripheral DCs can migrate into the thymus. Li et al. reported that two of the three major subsets of thymic DCs originate extrathymically and continually migrate to the thymus [28]. It has been demonstrated that BM‐derived antigen-presenting cells (APCs) carrying antigens from the periphery migrate into the thymus and delete autoreactive cells [29]. Roberto and his colleagues also reported that the homing of peripheral DCs to the thymus resulted in the clonal deletion of thymocytes [26]. Similarly, the CD8^lo^Sirpα^+^ DCs migrated from the periphery to the thymus and induced T-cell development and differentiation [30]. These findings further declared that circulating DCs migrated into the thymus and interacted with thymocytes. In this study, mDCs, generated from the GM-CSF and IL-4 induced bone marrow cells of EGFP^Tg/+^ mice, were intravenously injected into wild-type mice. Three days later, GFP^+^ CD11c^+^ cells were detected in the thymus of the recipient mice via FCM assay (Fig. 1). This result revealed that the mDCs were indeed able to return to the thymus. Homing DCs have been mainly reported to deplete thymocytes and induce tolerance. However, mTECs play a crucial role in inducing immune tolerance. Thus, we evaluated whether the mDCs homing into the thymus led to TECs depletion. We cocultured mDCs with mTEC1 cells and found that the mDCs induced the apoptosis and inhibited the proliferation of mTEC1 cells. These effects were only achieved via direct cell–cell contact between mDCs and mTEC1 cells (Fig. 2). Furthermore, we observed that an intrathymic injection of the mDCs resulted in acute thymic atrophy and reduced thymocytes and TECs substantially in vivo (Fig. 5). All these evidences demonstrated that circulating mDCs migrated into the thymus and induced the degeneration of the thymus.

Decreased expression of growth hormones and increased expression of sex hormones contribute to the degeneration of the thymus [31]. The involution process can be reversed after treatment with cytokines or other reagents such as keratinocyte growth factor [32, 33], IL-7 supplements [34, 35], or sex steroid ablation therapy [36], which can promote regeneration of TECs and restore the diversity of the peripheral TCR repertoire. However, the exact mechanisms underlying thymus degeneration and reconstitution remain poorly understood.

Notch signaling plays crucial roles in the development of the thymus and the differentiation of T cells. In addition, Notch signaling induces TECs development at an early phase of thymic organogenesis through the interaction between thymocytes and TECs [37]. It has been reported that mice, lacking the Notch ligand Jagged2, exhibited decreased medullary areas [17]. Deleting Notch1 in TECs resulted in the depletion of mTEC progenitors and substantially reduced the development of mTECs during fetal stages. In contrast, forced Notch signaling activation in all TECs resulted in the extensive expression of mTEC progenitor markers but profound defects in TECs differentiation [38]. Similarly, loss of Notch signaling functions resulted in mTECs hypoplasia. However, the maturation of TEC progenitors whose Notch signaling was defected would be inhibited after Notch signaling function was restored [10]. Goldfarb et al. confirmed that decreasing Notch signaling was required in the later stages of mTECs differentiation. TEC-specific overexpression of Notch1 reduced the populations of total TECs and mTECs [19]. Taken together, these findings demonstrated that the higher level of the Notch signaling is required in the early stages of thymus development, but a lower level of the Notch signaling is necessary for the homeostasis of TECs in the later stages. It was also proved that the mRNA expression of Notch3 was considerably higher in cTECs at later stages [39]. However, when examining the expression pattern of Notch proteins in the thymus, we found that the Notch3 protein was majorly expressed in mTECs, whereas Notch1 was primarily expressed in cTECs, and the expression levels in both cell types were decreased with aging (Fig. 3 and S1). These findings implied that Notch1 and Notch3 signaling contribute to the involution of cTECs and mTECs and also indicated that the Notch signaling is necessary for the initial development of the thymus. However, lower Notch signaling might maintain the stability and cellular homeostasis of TECs. To address these hypotheses, we used rmJagged1-hFc and DAPT as the Notch signaling activator and inhibitor, respectively, and incubated them with mDCs and mTEC1 cells. The results demonstrated that rmJagged1-hFc promoted cell apoptosis and inhibited cell proliferation of mTEC1 cells, and DAPT reversed these processes (Fig. 4). In addition, transfected with NICD3 to activate Notch signaling, the cell proliferation of mTEC1 was decreased (Fig. S2). Furthermore, the thymus developed acute atrophy after the intrathymic injection of rmJagged1-hFc, which was evidenced by the decreased numbers of thymocytes and TECs (Fig. 6).

Because Notch signaling activation requires receptor/ligand interactions, the expression of Notch ligands in the thymus is equally vital. The expression of the Notch ligand Jagged2 was steady during thymus development, while Jagged1 decreased from the embryonic to postnatal stages [40]. In addition, overexpression of Jagged1 on T cells could induce the apoptosis of TECs, resulting in thymic atrophy [18]. These findings suggested that the depressed expression of Jagged1 would contribute to the homeostasis of the thymus. Many investigations have elucidated that Jagged1, Jagged2, and DLL4 are expressed in DCs and that their expression levels are upregulated following the maturation of DCs induced by LPS stimulation [41–43]. We also observed that the expression of Jagged1 was increased in the mDCs after LPS stimulation (Fig. 3). This increased Jagged1 promoted the apoptosis and growth inhibition of TECs when they were cocultured with mDCs. In addition, DAPT, a γ-secretase inhibitor, attenuated these effects (Fig. 4).

**Fig. 7:**
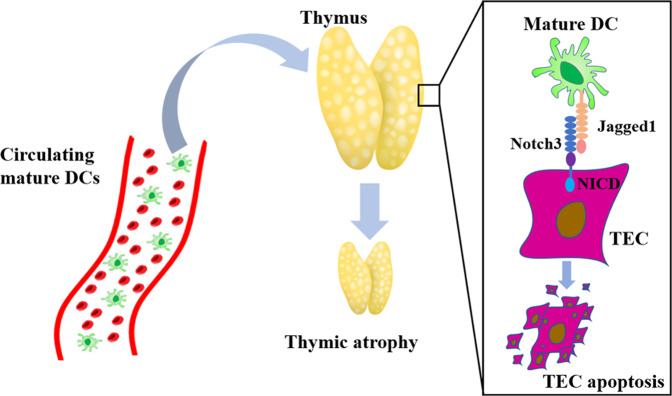
Research summary: circulating mature DCs homing to the thymus promote thymic epithelial cell involution through the Jagged1/Notch3 axis. Circulating mDCs can return to the thymus where they interact with TECs through direct cell–cell contact. MDCs can activate Notch signaling in TECs through the Jagged1/Notch3 axis. Long-term Notch signaling activation of TECs results in apoptosis and growth inhibition, further leading to the degeneration of the thymus.

Overall, the findings of this study improve our understanding of the mechanisms underlying thymus degeneration. During infection, activated DCs are mature, and migrate into different lymph nodes through afferent lymphatic vessels [44]. DCs, residing in tissues, can reach the periphery and carry antigens to secondary lymphoid organs through blood [45]. A small number of circulating DCs, capturing pathogens, can migrate into thymus [46]. Although the number of thymic homing DCs is relatively small, given numerous mild or severe infections throughout our lifetime, the cumulative effects may contribute to age-related thymus degeneration. In summary, our results provided evidence that circulating mDCs return to the thymus and interact directly with TECs to activate Notch signaling through the Jagged1/Notch3 axis (Fig. 7). Long-term Notch signaling activation of TECs results in their apoptosis and growth inhibition, thus leading to the degeneration of the thymus. These results also provide insights into the mechanisms underlying age-related thymic atrophy or infection, organ transplant rejection, and other diseases related acute thymic atrophy and help to develop novel strategies in clinical thymus and T-cell reconstruction.

## Materials and methods

### Animals

Male C57BL/6 (B6; H-2b) mice aged 2–3 weeks, 6–8 weeks, and 10 months were purchased from the Institute of Comparative Medicine, Yangzhou University (Yangzhou, P. R. China). EGFP^Tg/+^ mice were kindly donated by Dr. Xia Sheng (Department of Immunology, School of Medicine, Jiangsu University, Zhenjiang, Jiangsu, P. R. China).

### Cell lines and cell culture

mTEC1 cells were a kind gift from Prof. Weifeng Chen (Department of Immunology, Peking University Health Science Center, Beijing, P. R. China) [47]. L cells (a fibroblast cell line without Jagged1 expression) and J cells (a fibroblast cell line stably transfected with Jagged1) were provided by Dr. Xiaojie Zhang (Department of Neurology, University of Michigan, Ann Arbor, Michigan, USA). All cells were cultured in Dulbecco’s modified Eagle’s medium (DMEM) (Gibco, Thermo Fisher Scientific Shanghai Instruments Co. Ltd.) supplemented with 10% fetal bovine serum (Gibco), 100 U/ml penicillin, and 100 mg/ml streptomycin at 37 °C in a humidified atmosphere with 5% CO_2_.

### Reagents and antibodies

Recombinant murine GM-CSF and IL-4 were purchased from PeproTech China (#315–03, #214–14, Jiangsu, China). LPS was purchased from Sigma Aldrich, Merck Life Science (Cat#L2880, Shanghai, China). The following antibodies were used in FCM: CFSE (Cat#65-0850-84) anti-CD11c-cy5.5 (Cat#35-0114-82), anti-CD11b-APC (Cat#17-0112-82), anti-CD80-PE (Cat#12-0801-82), anti-CD86-PE (Cat#12-0862-82), anti-MHC Class II-FITC (Cat#11-5321-82) and anti-MoCD339-PE (Jagged1; Cat#12-3391-80, 1.25 μg/ml) were purchased from eBioscience, Thermo Fisher Scientific Shanghai Instruments Co. Ltd. (Shanghai, China). Anti-CD4-PE (Cat#100408), anti-CD8-APC (Cat#100712), anti-mouse Notch3-PE (Cat#130507), anti-mouse CD326-APC (Ep-CAM; Cat#118214), anti-mouse CD45-Alexa Fluor 488 (Cat#103122), and the APC Annexin V apoptosis detection kit with 7-AAD (Cat#640930) were purchased from Biolegend (CA, USA). The Annexin V-FITC/PI apoptosis detection kit was purchased from Multisciences (Lianke) Biotech Co., Ltd. (#70-AP101-100, Zhejiang, China). The recombinant mouse Jagged1-human Fc fusion protein (rmJagged1-hFc) was procured from Chimerigen Laboratories (Cat#CHI-MF-111JAG1, MA, USA). The following antibodies and reagents were used in the immunofluorescence assay: Hoechst 33258 staining dye solution (Cat#ab228550) and anti-Notch3 (Cat#ab23426) were purchased from Abcam Trading (Shanghai) Co. Ltd., Shanghai, China. In addition, we used DAPI (Cat# 28718-90-3, Cayman Chemical Co. Michigan, USA), anti-cytokeratin 8 (AB_531826, Developmental Studies Hybridoma Bank [DSHB], Iowa, USA), Alexa Fluor 488-labeled goat anti-rabbit IgG (H + L) (Cat# A0423, Shanghai Beyotime Biotechnology Co., Ltd., Shanghai, China), and Alexa Fluor 555 conjugated anti-rat IgG (H + L; Cat#4417, Cell Signaling Technology, Inc. MA, USA). DAPT was purchased from Selleck Chemicals (Cat#208255-80-5, TX, USA). The Cell-Light EdU Apollo567 in vitro kit was purchased from Guangzhou RiboBio Co., Ltd. (Cat# C10310-1, Guangzhou, China).

### Generation of BM-derived DCs

BMDCs were generated as described previously [48]. In brief, C57BL/6 bone marrow cells were isolated and cultured for 7 days in DMEM supplemented with 10% fetal bovine serum, GM-CSF (10 ng/ml), and IL-4 (1 ng/ml). Every 2 days, half of supernatant was replaced with fresh medium containing cytokines. On day 7, the suspended and aggregated cells were harvested. LPS (1 μg/ml) was added to promote BMDCs maturation.

### Flow cytometry analysis

All cells were harvested and incubated with rat serum to block Fc receptors. Subsequently, the cells were incubated with appropriate fluorescein‐conjugated antibodies for 30 min at 4 °C. After the cells were washed with precooled 1× phosphate-buffered saline (PBS), they were analyzed cells by using a CytoFLEX flow cytometer (Beckman Coulter, Inc. CA, USA). All FCM data were analyzed using FlowJo10.0 software.

### Cell coculture and transwell assay

The mTEC1 cells were seeded in a 24-well plate at a density of 2 × 10^4^ cells/well. After 6 h, imBMDCs or mBMDCs were added into the same wells at 8 × 10^4^ cells per well. The cells were continuously incubated for another 48 h. Subsequently, we examined all the cells through FCM or by performing a proliferation assay.

During the transwell assay, BMDCs were seeded in the upper layer of cell culture inserted with a permeable membrane at 3.2 × 10^5^ cells per well, and mTEC1 cells were placed in the bottom chamber under the membrane at 0.8 × 10^5^ cells per well. After incubation for 48 h, mTEC1 cells were harvested and stained for FCM analysis or proliferation assay.

### Cell apoptosis assay

Apoptosis was detected through staining with an Annexin V/PI apoptosis kit according to the manufacturer’s protocol. In brief, mTEC1 cells were collected and resuspended in 500 μl of binding buffer and incubated with 5 μl of Annexin V and 5 μl of PI or 7-AAD for 15 min at room temperature away from light. Apoptotic cells were analyzed by FCM.

### Cell proliferation assay

The proliferation of mTEC1 cells was examined by performing an EdU incorporation assay according to the manufacturer’s protocol. In brief, mTEC1 cells were seeded in a 24-well plate at a density of 2 × 10^4^ cells per well. After stimulation, EdU was added into the medium for 2 h. Then, 4% formaldehyde in PBS was used to fix cells for 30 min. Subsequently, 2 mg/ml glycine was used to stop fixation for 5 min. After washed with PBS, the cells were added Apollo staining buffer and incubated for 5 min. Finally, Hoechst 33258 was used to stain the cell nucleus. After washing with PBS, we scanned the stained cells under a fluorescence microscope. All steps were performed in dark.

### Intrathymic injection

Intrathymic injection was administered as described previously [49]. C57BL/6 mice were anesthetized with 50 mg/kg pentobarbital sodium through intraperitoneal injection and immobilized in supine recumbency on a surgical board. The chest skin was sheared and disinfected with povidone-iodine (PVP-I) solution. Subsequently, the skin between the lower neck and the upper thoracic region was cut along the sternum, and the chest wall was exposed. The upper one-third of the sternum was bisected to reveal the thymus. Each thymic lobe was injected with 10 μl of PBS containing 1.5 μg of mJagged1-hFc protein or 1 × 10^6^ BMDCs by using a 25-μl cuspidal Hamilton’s life science syringe. The thymic lobes of mice received the same volume of PBS as the control. The needle was withdrawn, and the skin and chest wall were closed with wound clips.

### Statistical analysis

Experimental data are presented as the mean ± standard error of the mean (all experiments were performed in triplicate). Statistical comparisons were performed using unpaired Student’s *t* test for two groups or analysis of variance for multiple groups. “*” represents *P* < 0.05, “**” represents *P* < 0.01, and “***” represents *P* < 0.001. All these values were deemed to indicate statistical significance, and ns indicates no significance. The figures are representative of three independent experiments.

## Supplementary information

Supplemental Information

Figure S1

Figure S2

## Data Availability

All original figures and data are deposited at https://pan.baidu.com/s/1Cn11R672Fyy-jwwoXIg0Eg with the password 1r0u.

## References

[1] Steinmann GG, Klaus B, Muller-Hermelink HK (1985). The involution of the ageing human thymic epithelium is independent of puberty. A morphometric study. Scand J Immunol.

[2] Chiang K, Kalantar-Zadeh K, Gupta A. Thymic dysfunction and atrophy in COVID-19 disease complicated by inflammation, malnutrition and cachexia. SSRN Electron J 2020. 10.1177/02601060221083160 PMC8891908 35234100

[3] Ye P, Kirschner DE, Kourtis AP (2004). The thymus during HIV disease: role in pathogenesis and in immune recovery. Curr HIV Res.

[4] Velardi E, Clave E, Arruda LCM, Benini F, Locatelli F, Toubert A (2021). The role of the thymus in allogeneic bone marrow transplantation and the recovery of the peripheral T-cell compartment. Semin Immunopathol.

[5] Vallejo AN, Michel JJ, Bale LK, Lemster BH, Borghesi L, Conover CA (2009). Resistance to age-dependent thymic atrophy in long-lived mice that are deficient in pregnancy-associated plasma protein A. Proc Natl Acad Sci USA.

[6] Shames RS (2002). Gender differences in the development and function of the immune system. J Adolesc Health.

[7] Kellogg C, Equils O (2020). The role of the thymus in COVID-19 disease severity: implications for antibody treatment and immunization. Hum Vaccin Immunother.

[8] Abramson J, Anderson G (2017). Thymic epithelial cells. Annu Rev Immunol.

[9] Hince M, Sakkal S, Vlahos K, Dudakov J, Boyd R, Chidgey A (2008). The role of sex steroids and gonadectomy in the control of thymic involution. Cell Immunol.

[10] Liu D, Kousa AI, O'Neil KE, Rouse P, Popis M, Farley AM, (2020). Canonical notch signaling controls the early thymic epithelial progenitor cell state and emergence of the medullary epithelial lineage in fetal thymus development. Development.

[11] Balciunaite G, Keller MP, Balciunaite E, Piali L, Zuklys S, Mathieu YD (2002). Wnt glycoproteins regulate the expression of FoxN1, the gene defective in nude mice. Nat Immunol.

[12] Soza-Ried C, Bleul CC, Schorpp M, Boehm T (2008). Maintenance of thymic epithelial phenotype requires extrinsic signals in mouse and zebrafish. J Immunol.

[13] Bray SJ (2016). Notch signalling in context. Nat Rev Mol Cell Biol.

[14] Siebel C, Lendahl U (2017). Notch signaling in development, tissue homeostasis, and disease. Physiol Rev.

[15] Radtke F, Fasnacht N, MacDonald HR (2010). Notch signaling in the immune system. Immunity.

[16] Meurette O, Mehlen P (2018). Notch Signaling in the Tumor Microenvironment. Cancer Cell.

[17] Jiang R, Lan Y, Chapman HD, Shawber C, Norton CR, Serreze DV (1998). Defects in limb, craniofacial, and thymic development in Jagged2 mutant mice. Genes Dev.

[18] Beverly LJ, Ascano JM, Capobianco AJ (2006). Expression of JAGGED1 in T-lymphocytes results in thymic involution by inducing apoptosis of thymic stromal epithelial cells. Genes Immun.

[19] Goldfarb Y, Kadouri N, Levi B, Sela A, Herzig Y, Cohen RN (2016). HDAC3 is a master regulator of mTEC development. Cell Rep.

[20] Yamamoto M, Sato S, Hemmi H, Sanjo H, Uematsu S, Kaisho T (2002). Essential role for TIRAP in activation of the signalling cascade shared by TLR2 and TLR4. Nature.

[21] Van Gool SW, Vandenberghe P, de Boer M, Ceuppens JL (1996). CD80, CD86 and CD40 provide accessory signals in a multiple-step T-cell activation model. Immunol Rev.

[22] Banchereau J, Briere F, Caux C, Davoust J, Lebecque S, Liu YJ (2000). Immunobiology of dendritic cells. Annu Rev Immunol.

[23] Lin CL, Huang HM, Hsieh CL, Fan CK, Lee YL (2019). Jagged1-expressing adenovirus-infected dendritic cells induce expansion of Foxp3(+) regulatory T cells and alleviate T helper type 2-mediated allergic asthma in mice. Immunology.

[24] Cahill EF, Tobin LM, Carty F, Mahon BP, English K (2015). Jagged-1 is required for the expansion of CD4+ CD25+ FoxP3+ regulatory T cells and tolerogenic dendritic cells by murine mesenchymal stromal cells. Stem Cell Res Ther.

[25] Kijima M, Yamaguchi T, Ishifune C, Maekawa Y, Koyanagi A, Yagita H (2008). Dendritic cell-mediated NK cell activation is controlled by Jagged2-Notch interaction. Proc Natl Acad Sci USA.

[26] Bonasio R, Scimone ML, Schaerli P, Grabie N, Lichtman AH, von Andrian UH (2006). Clonal deletion of thymocytes by circulating dendritic cells homing to the thymus. Nat Immunol.

[27] Brunk F, Augustin I, Meister M, Boutros M, Kyewski B (2015). Thymic epithelial cells are a nonredundant source of Wnt ligands for thymus development. J Immunol.

[28] Li J, Park J, Foss D, Goldschneider I (2009). Thymus-homing peripheral dendritic cells constitute two of the three major subsets of dendritic cells in the steady-state thymus. J Exp Med.

[29] Huseby ES, Sather B, Huseby PG, Goverman J (2001). Age-dependent T cell tolerance and autoimmunity to myelin basic protein. Immunity.

[30] Proietto AI, van Dommelen S, Wu L (2009). The impact of circulating dendritic cells on the development and differentiation of thymocytes. Immunol Cell Biol.

[31] Lynch HE, Goldberg GL, Chidgey A, Van den Brink MR, Boyd R, Sempowski GD (2009). Thymic involution and immune reconstitution. Trends Immunol.

[32] Rossi SW, Jeker LT, Ueno T, Kuse S, Keller MP, Zuklys S (2007). Keratinocyte growth factor (KGF) enhances postnatal T-cell development via enhancements in proliferation and function of thymic epithelial cells. Blood.

[33] Alpdogan O, Hubbard VM, Smith OM, Patel N, Lu S, Goldberg GL (2006). Keratinocyte growth factor (KGF) is required for postnatal thymic regeneration. Blood.

[34] Andrew D, Aspinall R (2001). Il-7 and not stem cell factor reverses both the increase in apoptosis and the decline in thymopoiesis seen in aged mice. J Immunol.

[35] Andrew D, Aspinall R (2001). Interleukin-7 and not stem cell factor reverses both the increase in apoptosis and the decline in thymopoiesis seen in aged mice. Mech Ageing Dev.

[36] Sutherland JS, Goldberg GL, Hammett MV, Uldrich AP, Berzins SP, Heng TS (2005). Activation of thymic regeneration in mice and humans following androgen blockade. J Immunol.

[37] Masuda K, Germeraad WT, Satoh R, Itoi M, Ikawa T, Minato N (2009). Notch activation in thymic epithelial cells induces development of thymic microenvironments. Mol Immunol.

[38] Li J, Gordon J, Chen ELY, Xiao S, Wu L, Zuniga-Pflucker JC, (2020). NOTCH1 signaling establishes the medullary thymic epithelial cell progenitor pool during mouse fetal development. Development.

[39] Kim KY, Lee G, Yoon M, Cho EH, Park CS, Kim MG (2015). Expression analyses revealed thymic stromal co-transporter/Slc46A2 is in stem cell populations and is a putative tumor suppressor. Mol Cells.

[40] Felli MP, Maroder M, Mitsiadis TA, Campese AF, Bellavia D, Vacca A (1999). Expression pattern of notch1, 2 and 3 and Jagged1 and 2 in lymphoid and stromal thymus components: distinct ligand-receptor interactions in intrathymic T cell development. Int Immunol.

[41] Yamaguchi E, Chiba S, Kumano K, Kunisato A, Takahashi T, Takahashi T (2002). Expression of Notch ligands, Jagged1, 2 and Delta1 in antigen presenting cells in mice. Immunol Lett.

[42] Elyaman W, Bradshaw EM, Wang Y, Oukka M, Kivisäkk P, Chiba S (2007). JAGGED1 and delta1 differentially regulate the outcome of experimental autoimmune encephalomyelitis. J Immunol.

[43] Amsen D, Blander JM, Lee GR, Tanigaki K, Honjo T, Flavell RA (2004). Instruction of distinct CD4 T helper cell fates by different notch ligands on antigen-presenting cells. Cell.

[44] Worbs T, Hammerschmidt SI, Forster R (2017). Dendritic cell migration in health and disease. Nat Rev Immunol.

[45] Balan S, Saxena M, Bhardwaj N (2019). Dendritic cell subsets and locations. Int Rev Cell Mol Biol.

[46] Bonasio R, von Andrian UH (2006). Generation, migration and function of circulating dendritic cells. Curr Opin Immunol.

[47] Liu ZG, Haelens A, Wuyts A, Struyf S, Pang XW, Proost P (1996). Isolation of a lymphocyte chemotactic factor produced by the murine thymic epithelial cell line MTEC1: identification as a 30 kDa glycosylated form of MCP-1. Eur Cytokine Netw.

[48] Qiang Y, Xu J, Yan C, Jin H, Xiao T, Yan N (2017). Butyrate and retinoic acid imprint mucosal-like dendritic cell development synergistically from bone marrow cells. Clin Exp Immunol.

[49] Manna S, Bhandoola A (2016). Intrathymic injection. Methods Mol Biol.

