# Supplementary material for: Circulating mature dendritic cells homing to the thymus promote thymic epithelial cells involution via the Jagged1/Notch3 axis
Source: Cell Death Discov. 2021 Aug 30;7:225. doi: 10.1038/s41420-021-00619-5 (PMC8404188; doi:10.1038/s41420-021-00619-5)
Supplement: Supplementary file 1 — Supplemental Information [file 41420_2021_619_MOESM1_ESM.docx]

**Supplemental Information**

**Circulating mature dendritic cells homing to the thymus promote thymic epithelial cells involution via Jagged1/Notch3 axis**

Haojie Wu ^1*^, Xiaohan Li ^1*^, Chen Zhou ^1*^, Qihong Yu ^1^, Shiyao Ge^1^, Zihui Pan^1^, Yangjing Zhao^1^, Sheng Xia ^1^, Xiaoming Zhou^2^, Xia Liu ^1^, Hui Wang ^1, §^, Qixiang Shao ^1,3, §^

**Figure S1. Notch1 predominant expressed by cTECs and was decreased with aging.**

The representative images of the expressions of Notch1 and cytokeratin 8 (K8, a cTEC marker, red fluorescence) in thymus from different aged mice (2-3 weeks old, 6-8 weeks old and 10 months old), were revealed by immunofluorescence microcopy. The paraffin sections of thymus, from different aged mice, were stained with primary rabbit anti-Notch1 and rat anti-cytokeratin 8, antibodies followed by secondary Alexa Fluor 488 labeled goat anti-rabbit and Alexa Fluor 555-labeled goat anti-rat antibodies respectively. At the end the sections were staining with the DAPI. Scale bars represent 100 µm. All data are from three independent experiments. Shown are representative figures.

**Figure S2. Overexpressing of NICD3 reduced the cell proliferation of mTEC1 cells.**

(A) mTEC1 cells were infected with retrovirus vector containing NICD3 and selected with puromycin (1μg/ml) subsequently. The cell proliferation was detected by an EdU incorporation assay. The red dots indicated the proliferating cells. Scale bars represent 100 µm. (B) The percentages of red dots in all cells were calculated according to panel A. Shown are representative figures. Data are represented as the mean ± SD of three independent experiments. * p < 0.05, ** p < 0.01, *** p < 0.001, compared with control group.
