# Supplementary figures and images for: Circulating mature dendritic cells homing to the thymus promote thymic epithelial cells involution via the Jagged1/Notch3 axis
Source: Cell Death Discov. 2021 Aug 30;7:225. doi: 10.1038/s41420-021-00619-5 (PMC8404188; doi:10.1038/s41420-021-00619-5)

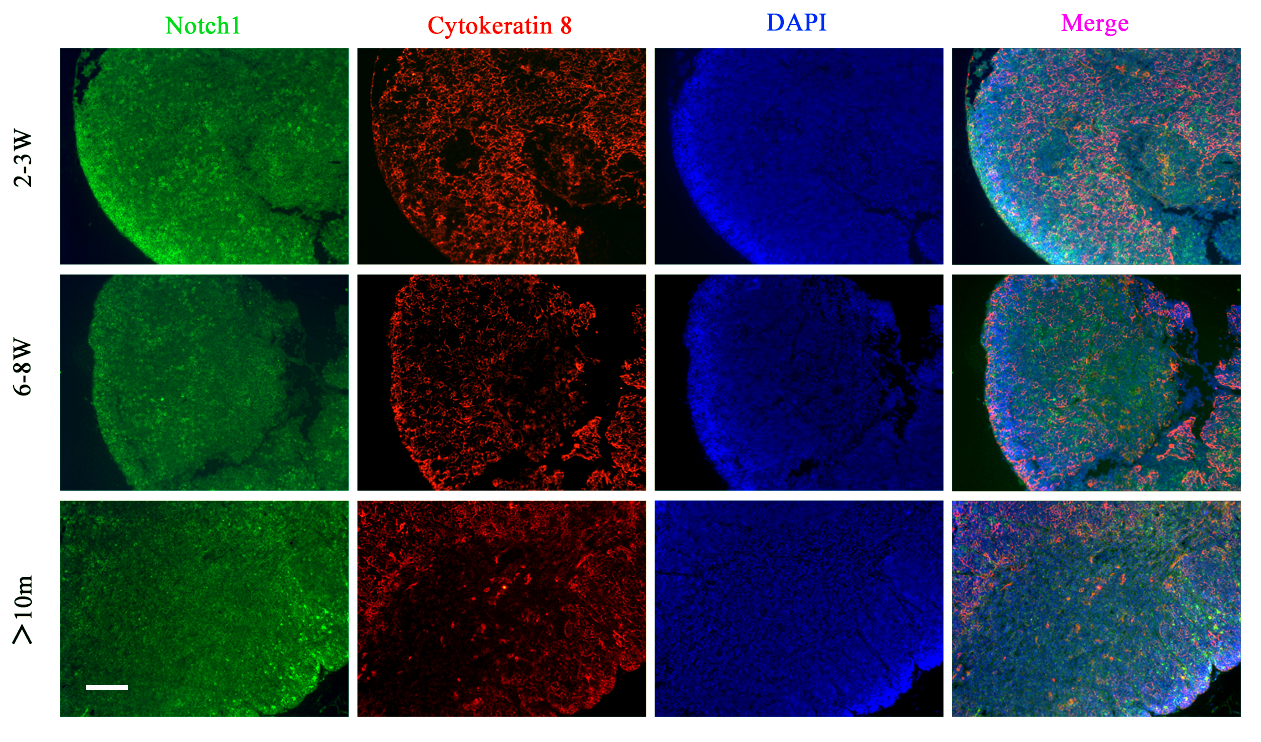

Supplement: Supplementary file 2 — Figure S1 [file 41420_2021_619_MOESM2_ESM.tif]

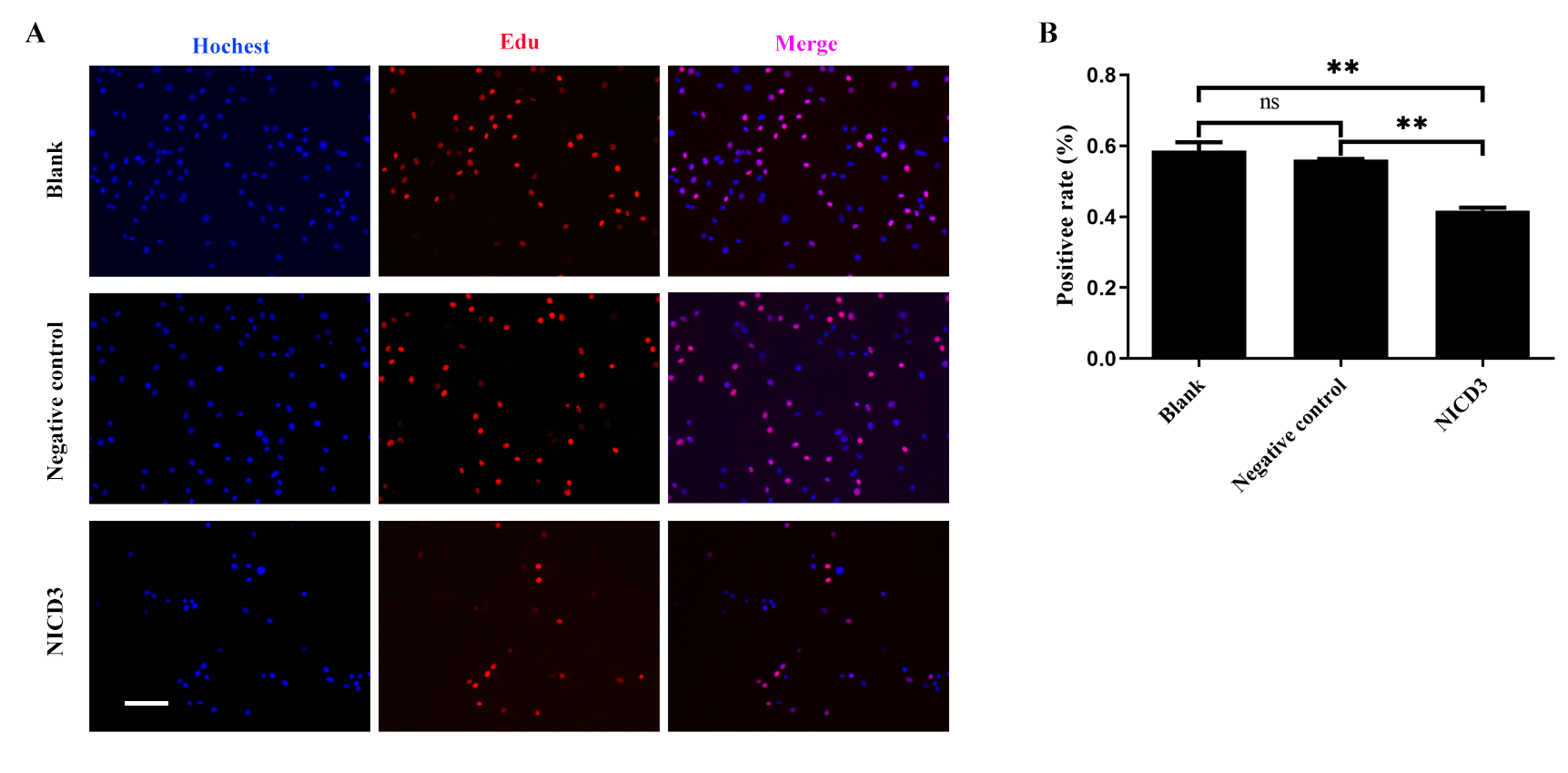

Supplement: Supplementary file 3 — Figure S2 [file 41420_2021_619_MOESM3_ESM.tif]
